# Nephritic Colic

**Published:** 1877-10

**Authors:** Ferdinand King

**Affiliations:** Atlanta, Ga.


### NEPHRITIC COLIC.

By FERDINAND KING, M.D., Atlanta, Ga.

I have recently attended a severe case of the above trouble, to which I wish to call the attention of the medical profession, on account of the successful result of the treatment, which, I must confess, was "accidental." The course pursued in the case I here report, may be familiar to some members of the profession, but I have not been able to find it in any text-book at my command.

I was called at one o'clock on the morning of May 10, 1877, to see Mr. O. A., a confectioner, on Marietta street, in this city, who, the messenger informed me, had been suffering since early in the night "with cramp colic." On reaching his apartment, I found the patient resting on his knees and elbows, in which position he expressed himself as being most comfortable, though the pain was constantly present, and confined to one side. It was of a sharp, lancinating character, beginning in the left lumbar region, and extending down through the left inguinal region, and along the spermatic cord to the left testicle. I at once decided that the case was one of *nephritic colic*, which is simply the result of the passage of a renal calculous, along the ureter, from the kidney to the bladder. Besides the pain referred to, the usual symptoms of disuria and increased frequency of micturition were also observed. At times, following intervals of short duration during which he was comparatively free from suffering, he was seized with the most intense pain, which he compared to the thought of being cut by a very dull knife. He was also suffering from extreme nausea, making strenuous efforts to vomit, but without success. I forgot to mention that the attack came on very suddenly, while the patient was attending a concert in the evening.

Morphine, subcutaneously, was the first remedy that suggested itself to me, but, as I had failed to provide myself with a suitable syringe before leaving my office, I was forced to adopt another form of administering the anodyne; so I gave half a grain of the salt in a teaspoonful of water, but it was immediately rejected by the stomach, which seemed to be in an extremely irritable condition. Thinking it necessary to improve that organ, by the use of an emetic, I gave my patient about thirty grains of ipecac. in half-glassful of cold water. To my utter surprise and astonishment, the medicine did not vomit him, dor did it even increase the nausea already present, but, on the other hand, it relieved the pain, as well as nausea, and the patient soon fell into a quiet, gentle sleep, that lasted about three quarters of an hour. On awaking, there was a recurrence of the pain, but of decidedly less severity. I then administered another thirty grains of ipecac., which again brought almost immediate relief, and a sleep that lasted two hours. As it was now about day-light, I gave him the third and last dose of ipecac.—thirty grains—making in all *ninety grains* of the drug. After this, he slept quietly until ten o'clock, when he awoke entirely free from pain, but very much prostrated. As he was badly constipated, the next object was to open his bowels. Having attended the gentleman in former troubles, (though of a different character) and knowing how difficult it was to accomplish this, I anticipated no cathartic effect from the large quantity of ipecac. I had given him, so I gave him four fluid ounces of cathartic elixir, which operated very copiously in the course of two hours.

During the remainder of the day he voided about a normal quantity of urine, which was of extremely high color, and slightly tinged with blood. I directed that it should be carefully preserved, so that I might find the "gravel," or renal calculus, when it came away. Late in the afternoon, I found in the bottom of the vessel in which the urine had been kept, a small calculus, which was mostly uric in its character, though the external covering, which was very rough, must have been of an ammonio-phosphatic character, as it soon crumbled off after exposure to the atmosphere, leaving only a small globular body, not more than half line in diameter.

Now, I would like very much to know the *modus operandi* by which the ipecac. so effectually relieved the patient. As I had given it for the purpose of cleaning out the stomach, so that another remedy might be borne, the discovery was, as before stated, purely accidental. Was the relief the result of relaxation or revulsion ? Or, was it from some unknown property resident in this valuable therapeutic agent ? I would also like to know why the large quantity of the drug administered did not produce vomiting ? The patient has since informed me that he had suffered from similar attacks before, but none so severe as the one just related. Said he never experienced more sudden and decided relief from taking any medicine in his life.

I hope my professional brethren will give the remedy a trial and report results.

